# Mitochondrial respiration and hydrogen peroxide production rate in right atrial tissues from obese diabetic patients

**DOI:** 10.1113/EP093121

**Published:** 2026-02-22

**Authors:** Toan Pham, Sarbjot Kaur, Anthony Hickey, Marie‐Louise Ward, Kenneth Tran

**Affiliations:** ^1^ Auckland Bioengineering Institute Auckland New Zealand; ^2^ Department of Physiology Faculty of Medical and Health Sciences Auckland; ^3^ School of Biological Sciences, Faculty of Science The University of Auckland Auckland New Zealand

**Keywords:** diabetic human heart, hydrogen peroxide production, mitochondrial respiration

## Abstract

Type 2 diabetes (T2D) is a global epidemic, with heart failure being the leading cause of premature death. Mitochondrial dysfunction, characterized by impaired energy metabolism, weakened energy transport system and increased oxidative stress, has been proposed as a key contributor to the impairment of contractile function in T2D hearts. However, direct evidence from human T2D hearts remains limited. We assessed the mitochondrial function of right atrial tissues obtained from consenting patients undergoing coronary bypass surgery, comparing T2D and non‐diabetic groups. We used high‐resolution respirometry and fluorometry techniques to assess mitochondrial O_2_ flux and H_2_O_2_ production in permeabilized cardiac fibres in simulated physiological conditions supported by ATP substrate. Our findings showed that O_2_ flux during oxidative phosphorylation and ATP‐stimulated respiration states was similar between groups. T2D fibres exhibited a lower H_2_O_2_ production rate during the leak state, both per tissue mass and per O_2_ consumed, but no group difference was observed in the oxidative phosphorylation respiratory state. These findings suggest that T2D, in the context of other comorbidities, such as coronary artery disease and obesity, might not contribute significantly to mitochondrial dysfunction in the human heart.

## Abstract (highlights)

- **What is the central question of this study?**
  How does type 2 diabetes (T2D) affect mitochondrial function and redox function in the human heart?
- **What are the main findings and their importance?**
  Using permeabilized right atrial tissues from T2D and non‐T2D patients, we examined mitochondrial respiration and hydrogen peroxide production in ATP–ADP channelling conditions. The O_2_ flux and H_2_O_2_ production were similar between groups. These findings suggest that, in the presence of other disease backgrounds, including coronary artery disease and obesity, T2D might not contribute significantly to mitochondrial dysfunction in human hearts.

## 1 INTRODUCTION

Cardiovascular complications are the leading cause of morbidity and mortality in patients with type 2 diabetes (T2D), and heart failure accounts for nearly 80% of fatalities (Boudina & Abel, 2007). The heart, a highly metabolic organ, depends on a constant supply of ATP, produced primarily by mitochondrial oxidative phosphorylation (OXPHOS). Mitochondrial dysfunction has been observed in T2D human hearts (Anderson et al., 2009; Croston et al., 2014; Ljubkovic et al., 2019), including decreased mitochondrial respiration, supported by malate and fatty acid substrates (Anderson et al., 2009, 2011; Montaigne et al., 2014), whereas respiration supported by carbohydrate‐derived substrates remains unchanged (Anderson et al., 2009, 2011; Ljubkovic et al., 2019; Montaigne et al., 2014), highlighting the substrate‐specific nature of mitochondrial dysfunction. Studies on animal models of diabetes have shown structural mitochondrial abnormalities (Boudina et al., 2007; Tang et al., 2023), impaired bioenergetic function (Boudina et al., 2007; Pham et al., 2014), increased production of reactive oxygen species (ROS) (Pham et al., 2014; Ye et al., 2004) and increased expression of mitochondrial uncoupling protein (Boudina et al., 2007), all of which contribute to decreased capacity for ATP synthesis (Pham et al., 2014; Ye et al., 2004).

In the heart, the creatine kinase (CK) shuttle system buffers ATP concentrations with contraction and facilitates ATP transport from mitochondria to contractile myofibrils in cardiac myocytes (Balaban, 2006). Mitochondrial CK rephosphorylates creatine within the mitochondrial intermembrane space using newly synthesized ATP, forming phosphocreatine, which diffuses to the myofibrils, where myofibrillar CK regenerates ATP from ADP. Studies on failing hypertrophic rat hearts, which model dilated cardiomyopathy, have reported increased mean distances between mitochondria and myofibrils with a defective CK system. These both would act to impair ATP–ADP exchange, thereby decreasing OXPHOS capacity and contraction at high workloads (Power et al., 2016, 2019). Diabetic rat hearts also show decreased mitochondrial CK protein expression (Jullig et al., 2007) and cardiac hypertrophy (Pham et al., 2025). T2D human hearts consistently show hypertrophy, with increased left ventricular wall thickness (Devereux et al., 2000) and increased atrial diameter (Wang et al., 2019). It remains unknown whether cardiac hypertrophy in human diabetic atrial tissues disrupts ADP diffusion and mitochondrial function in a similar manner.

In this study, we investigated mitochondrial respiration and H_2_O_2_ production rates under OXPHOS and ADP, channelling conditions in human heart right atrial tissues from T2D and non‐diabetic (ND) patients. We hypothesized that mitochondrial ADP transfer is impaired in T2D hearts, resulting in decreased mitochondrial respiration and increased H_2_O_2_ production. Using permeabilized fibres from the right atrial appendage of patients undergoing coronary artery bypass graft surgery, we implemented high‐resolution respirometry and fluorometry techniques to assess the effects of ADP diffusion on OXPHOS respiration while maintaining mitochondrial structure and cellular architecture.

## 2 MATERIALS AND METHODS

### 2.1 Ethical approval

This study was approved by the Human and Disability Ethics Committee of New Zealand (HDEC 17/STH/16) and conducted in accordance with the ethical standards laid down in the 2024 *Declaration of Helsinki*. Patients provided informed written consent before undergoing standard coronary artery bypass grafting surgery to provide a small sample of the right atrial appendage. De‐identified clinical records of patients donating tissues were obtained from Auckland City Hospital, Auckland, New Zealand.

### 2.2 Sample preparation

Right atrial appendage tissue samples from T2D patients (*n* = 14) and ND patients (*n* = 12) were collected immediately after excision by the surgeon and transported to our laboratory at the University of Auckland within 10 min in an oxygenated Krebs–Henseleit solution [in mmol/L: 118 NaCl, 4.75 KCl, 1.18 MgSO_4_.7 H_2_O, 1.18 KH_2_PO_4_, 24.8 NHCO_3_, 0.25 CaCl_2_, 10 glucose, and 20 2,3‐butanedione 2‐monoxime, bubbled with carbogen (95% O_2_ and 5% CO_2_) to maintain pH at 7.4]. Permeabilized fibres were used to evaluate mitochondrial function with several advantages, including: (1) a small tissue amount required for each experiment; (2) avoiding mitochondrial selection bias during the isolation procedure; and (3) maintaining mitochondrial networking structure for more physiological measurements.

### 2.3 Permeabilized fibres

After connective and adipose tissues were removed, fresh atrial tissue was mechanically teased apart into small bundles of fibres in ice‐cold BIOPS buffer (in mmol/L, 50 K‐MES, 30 sucrose, 20 taurine, 20 imidazole, 15 PCr, 7.23 K_2_EGTA, 2.77 CaK_2_EGTA, 6.56 MgCl_2_, 5.7 ATP and 0.5 dithiothreitol, with pH adjusted to 7.1 at 0°C) and permeabilized with 50 mg/L saponin for 30 min on ice. Fibres were then washed three times, each for 10 min, in ice‐cold MiRO5 buffer (in mmol/L: 110 sucrose, 60 potassium lactobionate, 20 HEPES, 20 taurine, 10 KH_2_PO_4_, 3 MgCl_2_, 0.5 EGTA and 1 g/L bovine serum albumin free fatty acid, with pH adjusted to 7.1 at 30°C), quickly blotted dry on filter paper and weighed for the mitochondrial experiments. An additional atrial sample (∼20 mg) was rapidly frozen at 80°C for enzyme activity analysis.

### 2.4 High‐resolution respirometry

Mitochondrial respiration was measured using a high‐resolution fluorespirometer (O2k, Oroboros Instruments, Innsbruck, Austria) with two independent 2 mL chambers. Each chamber included a polarographic oxygen sensor and a sealed stopper for substrate–inhibitor titration. Two detachable fluorimeters (Oroboros Instruments) were inserted in front of each chamber window to measure the dye fluorescence and O_2_ flux simultaneously. All measurements were conducted at 37°C. All substrates, uncouplers and inhibitors were added manually with Hamilton syringes. Two titration protocols were used in this study.

### 2.5 Protocol 1: ADP channelling system

Permeabilized fibres (∼2 mg) were added to each chamber with 2 mL MiRO5 buffer, and the O_2_ concentration was kept in the range of 300–500 µmol/L to overcome O_2_ diffusion barriers in the fibre preparation. The substrates, uncoupler and inhibitors were added in sequential order. Malate (2 mmol/L), glutamate (10 mmol/L) and pyruvate (5 mmol/L) were added to activate complex I (CI) leak state. Succinate (10 mmol/L) was used to quantify leak state from CI‐ and complex II (CII)‐supported electron inputs. ATP (5 mmol/L) was added to activate endogenous OXPHOS respiration. Phosphoenolpyruvate (2 mmol/L) and pyruvate kinase (10 U) were used to trap ADP, and creatine (10 mmol/L) was used to promote localized ADP production. The uncoupler carbonyl cyanide *m*‐chlorophenyl hydrazone (CCCP, 1 µmol/L) was used to measure uncoupled respiration as an estimate of maximal electron transport system capacity. Antimycin A (1 µmol/L) was added to inhibit complex III and measure residual O_2_ consumption.

### 2.6 Protocol 2: Maximal OXPHOS respiration

Once permeabilized fibres were added to a measurement chamber, the leak state was initiated with malate, glutamate, pyruvate and succinate as above. ADP (2.5 mmol/L) was added to stimulate CI and CII OXPHOS. CCCP (1 µmol/L) was used to measure uncoupled respiration. Antimycin A (1 µmol/L) was added to inhibit complex III to determine residual O_2_ consumption.

### 2.7 Measurements of H_2_O_2_ production rate

The H_2_O_2_ production rate was measured simultaneously with O_2_ flux during titration Protocol 1 using Amplex UltraRed (Thermo Fisher, USA) in the presence of horseradish peroxidase and superoxide dismutase using Protocol 1. The addition of exogenous superoxide dismutase reduces superoxide radicals produced from mitochondria to form H_2_O_2_. The combined mitochondrial and exogenous superoxide dismutase‐derived H_2_O_2_ is coupled to horseradish peroxidase, which reacts with Amplex Ultrared to create resorufin, a fluorescent molecule with excitation and emission wavelengths of 525 and 550 nm, respectively (Pham et al., 2020). Amplex UltraRed (25 µmol/L), horseradish peroxidase (10 U) and superoxide dismutase (10 U) were added to the chambers. To calibrate the H_2_O_2_ signal, each chamber was titrated three times with 0.122 µmol/L H_2_O_2_ and allowed to equilibrate before adding the samples.

### 2.8 Citrate synthase activity

A subset of frozen muscle tissues stored at −80°C were thawed, weighed (∼30 mg), and gently minced with scissors in a 10‐fold volume of cold homogenization buffer (in mmol/L: 1 EDTA, 2 MgCl_2_, 50 KCl, 25 Tris–HCl and 0.5% Triton X‐100 at pH 7.8). Samples were homogenized using a tissue lyser at 30 Hz for 30 s, then centrifuged at 14 000*g* for 10 min at 4°C. The supernatants were collected to assess citrate synthase (CS) activities, which were measured at 412 nm and 25°C in a plate reader in the presence of 0.5 mmol/L oxaloacetate, 0.1 mmol/L acetyl‐CoA and 0.2 mmol/L 5,5‐dithiobis(2‐nitrobenzoic acid) in 50 mmol/L Tris–HCl buffer (pH 8). CS activity was calculated using an extinction coefficient of 13.6 L/mmol/cm and normalized to the corresponding protein concentration, determined by Bicinchoninic Acid Protein assay (Thermo Fisher Scientific, USA).

### 2.9 Data analyses

Respirometric and fluorometric data were recorded and analysed offline using DatLab v.7.1 (Oroboros Instruments). The O_2_ flux was corrected for residual O_2_ flux after antimycin addition. The H_2_O_2_ signals were corrected for background levels before sample addition. All data were normalized to tissue wet mass.

### 2.10 Statistical analyses

Statistical analyses were performed using GraphPad Prism v.10. Two‐way ANOVA with Fisher's least significant difference *post hoc* test was used to assess the interaction between groups and respiratory states. Student's unpaired *t*‐tests were used for comparison of a variable between the two groups. Data are presented as the mean ± SD. Statistical significance was set at *P* < 0.05. GraphPad was used to create the graphs.

## 3 RESULTS

### 3.1 Patient clinical data

Table 1 summarizes the demographic and clinical characteristics of the two patient groups. Patients were classified as T2D if their glycated haemoglobin (HbA1c) level was >40 mmol/mol and ND if their HbA1c level was <40 mmol/mol. Atrial tissues were obtained from 14 ND patients and 12 T2D patients. There was no significant difference in age (*P* = 0.3025) or body mass index (*P* = 0.4209) between the groups, and both cohorts had body mass index values of >28 kg/m^2^, considered overweight or obese.

**TABLE 1:** Characteristics of the two patient cohorts

Characteristic	Non‐diabetic (*n* = 14)	Diabetic (*n* = 12)
Age, years	68.2 ± 9.7	61.8 ± 14.4
Sex, male/female	14/0	11/01
BMI, kg/m^2^	29.8 ± 4.7	28.7 ± 3.6
Cigarette smoker, *n* (%)	4 (28.6)	4 (33.3)
Atrial fibrillation, *n* (%)	2 (14.3)	0
Hypertension, *n* (%)	8 (57.1)	9 (75)
Hyperlipidaemia, *n* (%)	8 (57.1)	10 (83.3)
Previous cardiac surgeries, *n* (%)	1 (7.1)	0
Pre‐operative medications, *n* (%)		
ACE inhibitors	10 (71.4)	6 (50)
AT1 blockers	1 (14.3)	3 (25)
B‐blockers	12 (85.7)	8 (66.7)
Ca^2+^ channel blockers	1 (7.1)	6 (50)
Diuretics	2 (14.3)	1 (8.3)
Nitrates	3 (21.4)	1 (8.3)
Lipid‐lowering drugs	14 (100)	12 (100)

*Note*: Values are expressed as the mean ± SD or *n* (%). Abbreviations: ACE, angiotensin‐converting enzyme; AT1, angiotensin II receptor type 1; BMI, body mass index.

### 3.2 Mitochondrial respiration in permeabilized fibres

**FIGURE 1:**
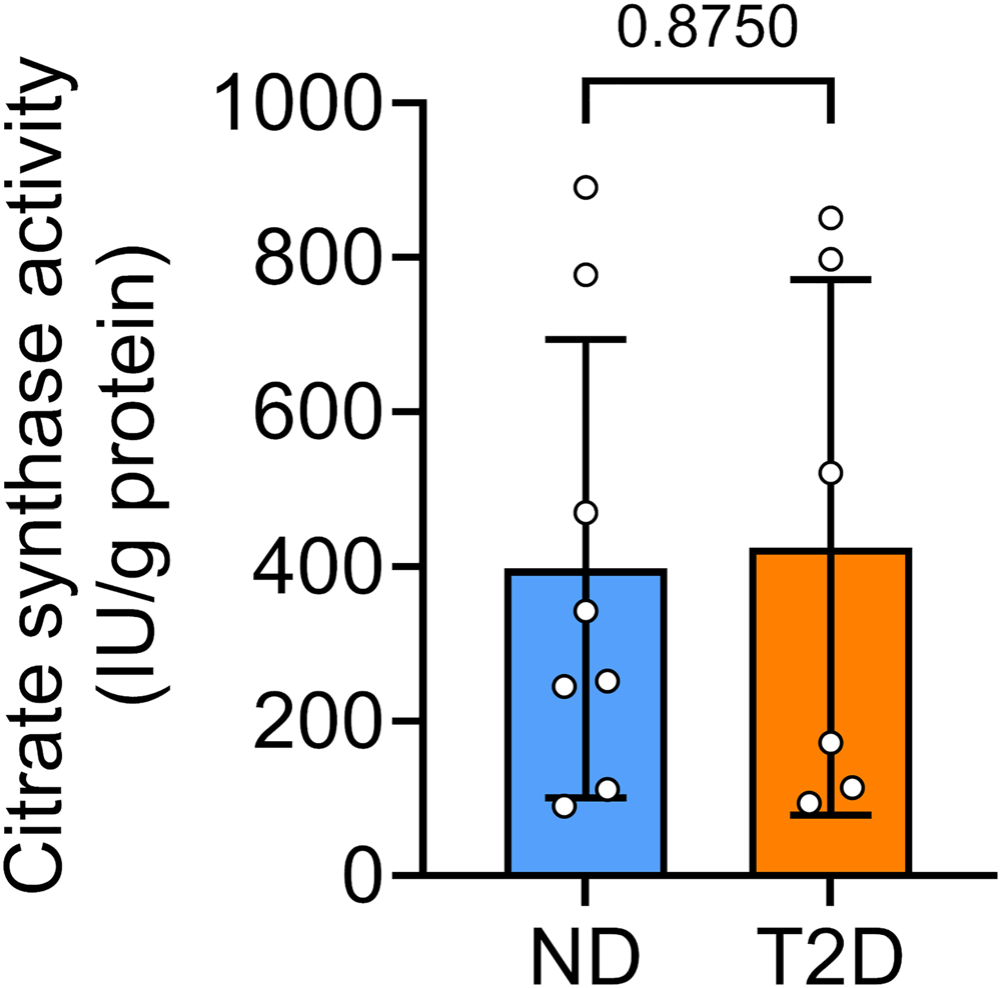
Citrate synthase activity normalized to total protein content. ND, *n* = 8; T2D, *n* = 6. Values are mean ± SD. Abbreviations: IU, micromoles per minute; ND, non‐diabetic; T2D, type 2 diabetic.

**FIGURE 2:**
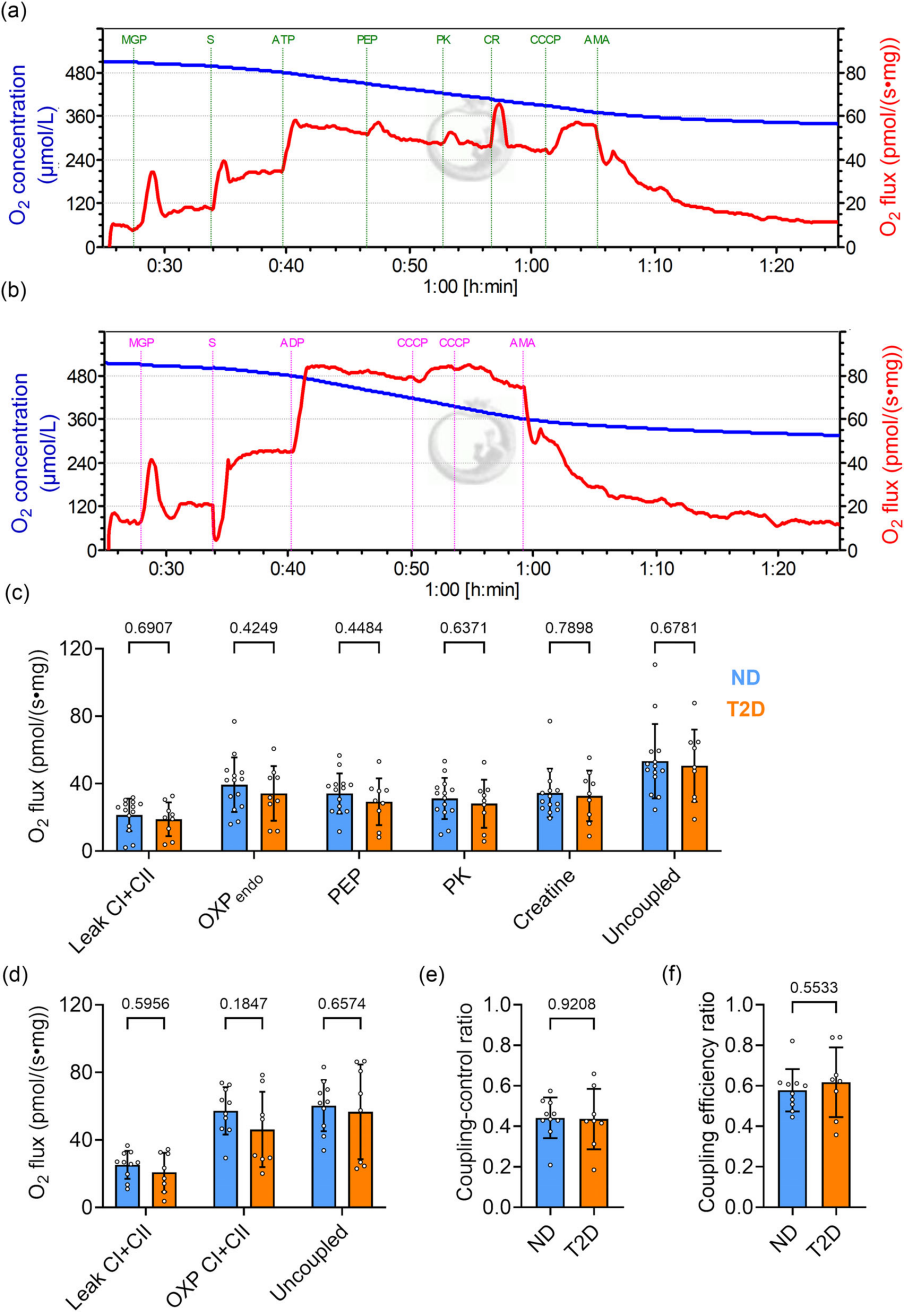
Mitochondrial respiration normalized to tissue mass in permeabilized atrial fibres. (a, b) Representative raw data traces showing O_2_ concentration (blue) and O_2_ flux (red) from Protocol 1 (a) and Protocol 2 (b). (c, d) Averaged O_2_ flux per tissue mass from Protocol 1 (c) and Protocol 2 (d) in various respiration states. (e) Coupling control ratio: leak respiration/OXPHOS respiration. (f) Coupling efficiency ratio: (uncoupled respiration − leak respiration)/uncoupled respiration. ND, *n* = 12–14; T2D *n* = 8–9. Values are the mean ± SD. Abbreviations: ADP, adenosine diphosphate; AMA, antimysin A; ATP, adenosine triphosphate; CCCP, carbonyl cyanide m‐chlorophenylhydrazone; CR, creatine; CI, complex I; CII, complex II; MGP, malate + glutamate + pyruvate; ND, non‐diabetic; OXP_endo_, endogenous OXPHOS respiratory state; PEP, phosphoenolpyruvate; PK, pyruvate kinase enzyme; S, succinate; T2D, type 2 diabetic.

There was no significant difference in CS activities between the groups (*P* = 0.8750; Figure 1), indicating similar mitochondrial content. In Protocol 1, we measured mitochondrial O_2_ flux and H_2_O_2_ production in permeabilized fibres to evaluate the ADP diffusion between mitochondria and myofibrils (Figure 2a). Leak respiration using CI and CII substrates is a respiration condition in which mitochondria consume O_2_ via electrons donated to CI and CII to compensate for the proton leak when ATP synthase is not active. In the presence of CI (malate, glutamate and pyruvate) and CII (succinate) substrates, ATP was used to drive the endogenous OXPHOS. This created favourable conditions for maintaining ATP production without immediately depleting ADP, thereby allowing us to evaluate the efficiency and capacity of OXPHOS. The O_2_ flux decreased after the addition of pyruvate kinase to catalyse the conversion of phosphoenolpyruvate to pyruvate while maintaining a high ATP/ADP ratio by trapping ADP at low levels, hence simulating restricted ADP diffusion. Creatine was added to regenerate ADP locally via mitochondrial CK, thus stimulating OXPHOS. When normalized to tissue mass, mitochondrial O_2_ flux showed no difference between groups in all respiratory states (*P* > 0.4249; Figure 2c).

In Protocol 2, we assessed mitochondrial OXPHOS respiratory capacity using a saturating ADP concentration. A protonophore uncoupler, CCCP, was used to drive maximal O_2_ flux during uncoupled respiration, in which no ATP is produced. Mitochondrial O_2_ flux was not different between groups, regardless of leak, OXPHOS or uncoupled respiratory states (*P* = 0.5956, *P* = 0.1847 and *P* = 0.6574, respectively; Figure 2d). The coupling control ratio (*P* = 0.9208; Figure 2e) and coupling efficiency ratio (*P* = 0.5533; Figure 2f) did not differ between groups.

### 3.3 H_2_O_2_ production rates in permeabilized fibres

**FIGURE 3:**
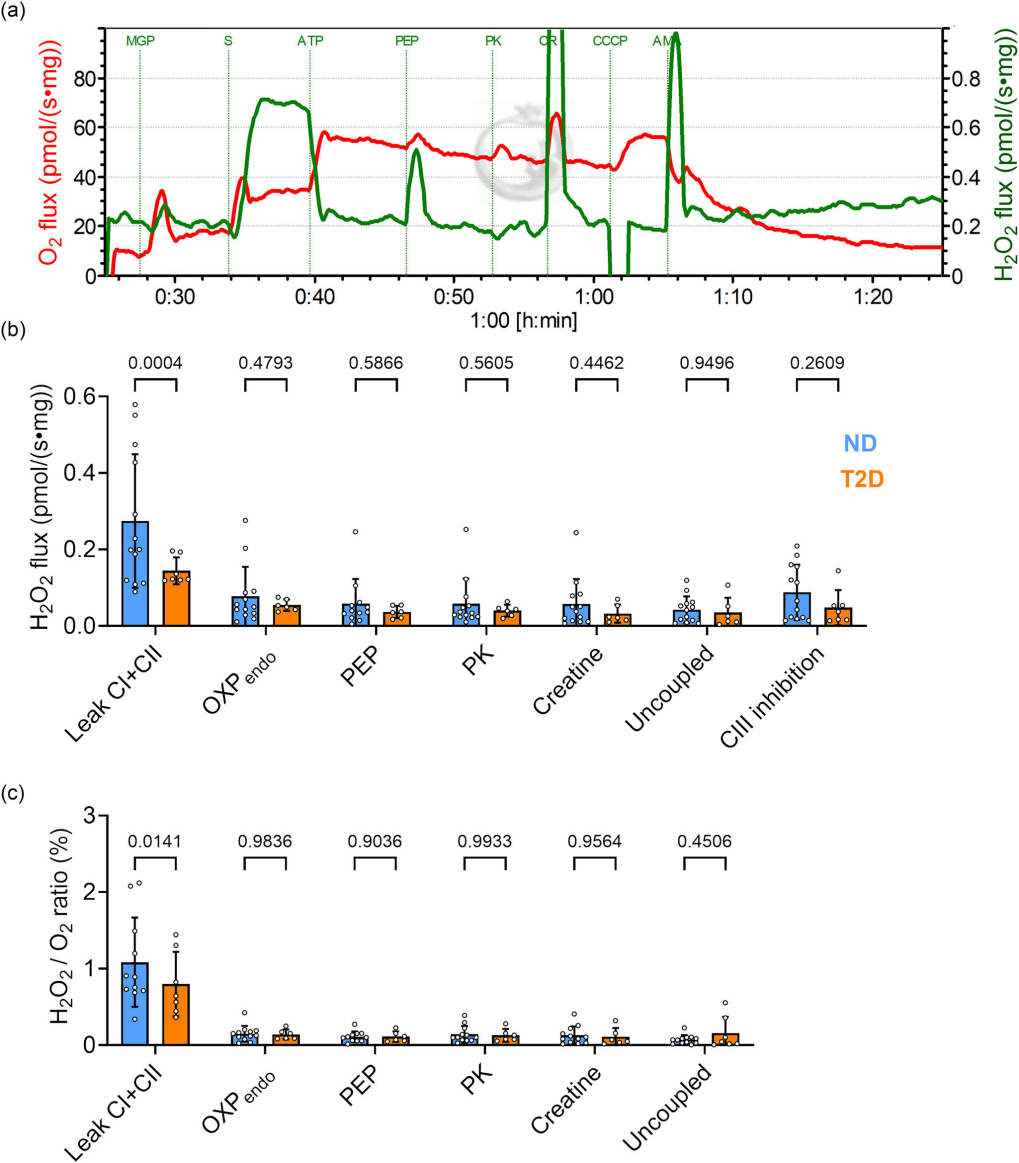
Mitochondrial H_2_O_2_ production rates from permeabilized fibres. (a) Representative raw data traces of H_2_O_2_ production rate (green) and O_2_ flux (red) from Protocol 1. (b) Net rates of H_2_O_2_ production normalized to tissue wet mass. (c) H_2_O_2_ flux relative to O_2_ flux (expressed as a percentage). ND, *n* = 13; T2D, *n* = 6. Values are the mean ± SD. *P < 0.05. Abbreviations: AMA, antimysin A; ATP, adenosine triphosphate; CCCP, carbonyl cyanide m‐chlorophenylhydrazone; CR, creatine; CI, complex I; CII, complex II; MGP, malate + glutamate + pyruvate; ND, non‐diabetic; OXP_endo_, endogenous OXPHOS respiratory state; PEP, phosphoenolpyruvate; PK, pyruvate kinase enzyme; S, succinate; T2D, type 2 diabetic.

Using fluorometry with Amplex Ultrared, we measured the net H_2_O_2_ production rate and O_2_ flux simultaneously (Figure 3a). The highest H_2_O_2_ production rate occurred in CI and CII leak state following succinate addition, probably owing to reverse electron transport at CI. When normalized to tissue mass (Figure 3b) or relative O_2_ flux (Figure 3c), the H_2_O_2_ production rate was found to be significantly higher in the ND group in CI and CII leak state (*P* = 0.004 and *P* = 0.0141, respectively), whereas no significant difference was observed in all other respiratory states between groups (*P* > 0.2609). The H_2_O_2_ production accounted for only 1%–2% of O_2_ consumption rate in the leak state and 0.2% during OXPHOS (Figure 3b).

## 4 DISCUSSION

To our knowledge, this is the first study to investigate mitochondrial respiration and H_2_O_2_ production in ADP channelling conditions in permeabilized human atrial fibres from diabetic and non‐diabetic patients. We found no significant differences in mitochondrial O_2_ flux between groups across various respiratory states. Interestingly, mitochondrial H_2_O_2_ production rate in the non‐phosphorylating leak state was higher in the non‐diabetic group, but unchanged in other respiratory states.

In this study, we supplied saturating concentrations of carbohydrate‐derived substrates to replicate a high‐workload environment in the cardiomyocytes. Our findings align with previous reports demonstrating no significant difference in mitochondrial O_2_ flux during OXPHOS, supported by combined CI and CII substrates (Anderson et al., 2009, 2011; Ljubkovic et al., 2019; Montaigne et al., 2014) and unchanged CS activity as an indicator of mitochondrial content in atrial tissues from T2D patients (Anderson et al., 2011; Croston et al., 2014; Duicu et al., 2016; Montaigne et al., 2014). Previous work using isolated subsarcolemmal and interfibrillar mitochondria from human atrial tissue found a selective decrease in CI and fatty acid‐supported mitochondrial respiration in subsarcolemmal mitochondria from T2D patients, but not in interfibrillar mitochondria (Croston et al., 2014), indicating that mitochondrial dysfunction is region specific in diabetic hearts. Although our study did not assess fatty acid‐supported respiration, other studies consistently report decreased mitochondrial respiration using fatty acid substrates in right atrial tissue from T2D patients (Anderson et al., 2009, 2011; Montaigne et al., 2014), suggesting that impaired utilization of fatty acid, despite its abundance in diabetic hearts (Ljubkovic et al., 2019), might underlie mitochondrial dysfunction.

Importantly, tissue samples from this study were all from obese patients with coronary artery disease (CAD), and many of them were on various medications. These comorbidities might independently affect mitochondrial function. Evidence shows that both obesity and CAD impair mitochondrial biogenesis and complex I activity in the human heart (Ait‐Aissa et al., 2019; Niemann et al., 2011). A recent study comparing mitochondria in right atrial appendages from patients with CAD, with and without T2D, found decreased OXPHOS and uncoupled respiration rates supported by carbohydrate‐derived substrates in CAD patients (Duicu et al., 2016). No additional mitochondrial impairment was observed in those patients with T2D. These findings support the interpretation that mitochondrial dysfunction in diabetic hearts is caused primarily by CAD and obesity, with a less significant contribution from diabetes.

Our study assessed the interaction between mitochondria and myofibrils and the impact of ADP channelling on O_2_ flux and ROS production. Endogenous OXPHOS respiration was stimulated by the addition of ATP, requiring ATP hydrolysis by cellular ATPases and subsequent ADP diffusion back into mitochondria. The PK/PEP system was used to trap cytosolic ADP, while creatine stimulated ADP regeneration via the mitochondrial CK system. This approach mimics the creatine phosphate shuttle system, facilitating high‐energy phosphate transfer in the heart. In diseased rat hearts, decreased CK activity and increased diffusion of ADP–ATP exchange were associated with decreased mitochondrial respiration and increased ROS production (Power et al., 2016). Our findings show no difference in respiration and H_2_O_2_ production in the ADP‐trapping system, suggesting that diabetes does not negatively impact mitochondrial function in T2D hearts, in addition to impairment attributable to other disease backgrounds.

### 4.1 ROS production

We measured mitochondrial H_2_O_2_ production rate and O_2_ flux simultaneously during various respiratory states in real time. Our data showing mitochondrial H_2_O_2_ production rate per O_2_ to be between 0.1 % and 3% are consistent with previous reports in diabetic rat hearts (Pham et al., 2014), hypertensive failing rat hearts (Power et al., 2016) or human skeletal muscle (Pham et al., 2020). The CI and CII leak state presents conditions of high succinate and limited ADP, mimicking ischaemic conditions in vivo. In this state, H_2_O_2_ production increased rapidly, probably owing to reverse electron transport to form superoxide at CI (Murphy, 2009). In these conditions, the T2D group showed a lower H_2_O_2_ production rate per tissue mass and per O_2_ consumed, consistent with our earlier study on streptozotocin‐induced diabetic rat hearts (Pham et al., 2014). These findings are in contrast to a previous study showing a higher mitochondrial H_2_O_2_ production rate in right atrial tissues from T2D patients (Anderson et al., 2011). The differences in experimental design, such as the use of palmitoyl‐l‐carnitine and a low concentration of ADP, might account for these discrepancies.

### 4.2 Limitations

This study has two limitations. First, the exclusive use of atrial tissues in this study might not be representative of the whole human heart. In particular, several structural and functional variations exist between atrial and ventricular mitochondria (Fang et al., 2026; Scheiber et al., 2019), which might influence their responses to pathological stimuli, such as diabetes or obesity. Second, we did not explore mitochondrial ADP channelling supported by fatty acid substrates. Future studies incorporating fatty acid metabolism are warranted to gain a better understanding of the bioenergetic consequences of diabetes.

## 5 CONCLUSION

Mitochondrial OXPHOS respiration and the ADP channelling appear to be preserved in atrial tissue from diabetic patients. Although non‐diabetic mitochondria generated more ROS output during ischaemia‐mimicking leak respiration, ROS output did not differ between groups during physiological respiration states. These findings suggest that diabetes does not significantly impact mitochondrial respiratory function or redox balance in the human heart, even in the presence of obesity and CAD.

## AUTHOR CONTRIBUTIONS

Toan Pham and Kenneth Tran designed the study. Toan Pham and Sarbjot Kaur performed the experiments. Toan Pham was responsible for statistical data analysis, preparing figures and drafting the manuscript. All authors contributed to the data interpretation, approved the final version of the manuscript and agree ‐ to be accountable for all aspects of the work in ensuring that questions related to the accuracy or integrity of any part of the work are appropriately investigated and resolved. All persons designated as authors qualify for authorship, and all those who qualify for authorship are listed.

## CONFLICT OF INTEREST

None declared.

## Data Availability

The data that support the findings of this study will be made available from the corresponding author upon request.
